# A Systematic Comparison of Purification and Normalization Protocols for Quantitative MicroRNA Expressional Profiling in Insulin-Producing Cells

**DOI:** 10.3390/ijms17060896

**Published:** 2016-06-07

**Authors:** Anna Lindeløv Vestergaard, Maaike Blankestijn, Jonathan Lucien Stahl, Emil Marek Heymans Pallesen, Claus Heiner Bang-Berthelsen, Flemming Pociot, Guy Wayne Novotny, Morten Lundh, Thomas Mandrup-Poulsen

**Affiliations:** 1 Laboratory for Immuno-Endocrinology, Department of Biomedical Sciences, University of Copenhagen, 2200 Copenhagen N, Denmark; m.blankestijn@umcg.nl (M.B.); jonathan@sund.ku.dk (J.L.S.); epallesen@sund.ku.dk (E.M.H.P.); guy_novotny@runbox.com (G.W.N.); lundh@sund.ku.dk (M.L.); tmpo@sund.ku.dk (T.M.-P.); 2 Center for Non-coding RNA in Technology and Health, Department of Pediatrics, Herlev and Gentofte Hospital, 2730 Herlev, Denmark; cbberthelsen@gmail.com (C.H.B.-B.); flemming.pociot.01@regionh.dk (F.P.); 3 Faculty of Health and Medical Sciences, University of Copenhagen, 2200 Copenhagen N, Denmark

**Keywords:** method, microRNA, purification, qPCR, quantification, normalization, pancreas

## Abstract

As microRNAs (miRs) are gaining increasing attention as key regulators of cellular processes, expressional quantification is widely applied. However, in the processing of relatively quantified data, the importance of testing the stability of several reference mRNAs and/or miRs and choosing among these for normalization is often overlooked, potentially leading to biased results. Here, we have optimized the purification of miR-enriched total RNA from pancreatic insulin-producing INS-1 cells. Additionally, we optimized and analyzed miR expression by a qPCR-based microarray and by specific qPCR and tested the stability of candidate reference mRNAs and miRs. Hence, this study gives a widely applicable example on how to easily and systematically test and decide how to normalize miR quantification. We suggest that caution in the interpretation of miR quantification studies that do not comprise stability analysis should be exerted.

## 1. Introduction

Characterizing the expressional levels of microRNA (miR) in various tissues and cell lines, and under different conditions, is increasingly performed by microarrays and quantitative PCR (qPCR) on purified miR [1]. The choice of one or more housekeeping genes or miRs for normalization of qPCR data is crucial to avoid potential technical bias. The choice of suitable mRNAs or miRs for normalization should be carefully selected and validated for the specific sample type and experimental conditions to ensure the stable expression of these between sample groups and thereby correct normalization and data processing [2,3,4]. Here, we use miR quantification from the insulin-producing cell line INS-1 to illustrate the importance of systematic optimization of RNA purification and quantitation of miRs from a specific sample type, including profiling and selection of suitable reference candidates.

The expression of miRs in pancreatic β cells is heavily influenced by a number of conditions [5,6,7,8]. In several studies on miRs in pancreatic β cells, traditionally used housekeeping genes, such as U6 and RNU6B, have been applied as endogenous controls for normalization, notably without any given justification for the choice of reference [9,10,11,12]. If quantification data are normalized to a reference gene which has not been tested for stability, this gene might vary systematically and hence introduce bias in the results. The present study is to our knowledge the first published systematic optimization of miR quantification in pancreatic β cells.

## 2. Results

### 2.1. Purification of miR-Enriched RNA from Pancreatic β Cells

We wished to characterize miR expression in pancreatic β-cells under different conditions and chose the widely used pancreatic rat insulinoma β-cell line INS-1 [13] for this purpose. We took advantage of INS-1 cells stably transfected with lentivirally transduced short hairpin RNA (shRNA) knockdown of histone deacetylases HDAC1, HDAC2, HDAC3, or empty vector (EV) shown previously to affect responses to inflammatory and metabolic stress [14,15], since knockdown of these key transcriptional regulators were expected to modify miR expression. These four different cell lines were harvested following exposure to interleukin-1 (IL-1) β and interferon (IFN) γ, known to affect both miR expression and mRNA expression of HDACs [11,16].

In order to obtain the best possible sample material, we compared the yield and quality of miR-enriched RNA obtained from four samples of INS-1 cells with three different purification kits. All three kits purify miR-enriched total RNA, enabling integrated analysis of mRNA from the same samples. The miRCURY cell and plant kit (Exiqon, Vedbaek, Denmark) was superior in yield and RNA purity and quality to the kits NucleoSpin miRNA (Macheray–Nagel, Düren, Germany) and miRNeasy (Qiagen, Hilden, Germany) (Table 1), and the miRCURY kit was therefore chosen. Moreover, the kit was the easiest and quickest to use, and it did not, unlike the miRNeasy kit, entail use of any organic solvents.

### 2.2. Stability Analysis of Candidates for Normalization of qPCR-Based miR Array

To map the global repertoire of β-cell miR expression, RNA samples were subjected to a SYBR Green qPCR-based microRNA array, detecting 752 miRs and 6 suggested references. To evaluate the stability of the given references, their expression was analyzed with the NormFinder algorithm plugin for Microsoft Excel [2]. NormFinder ranks a set of candidate mRNAs or miRs for optimal normalization according to their expression stability in a given sample set and given experimental design. When subjecting non-normalized expression values (2^−*C*t^) to analysis by NormFinder, one must designate to which group each sample belongs, the goal being to evaluate the stability across these groups. In this case, we had two options of grouping samples, that is, according to the transduced cell line (HDAC1, −2, −3 knockdown or EV) or exposure (with or without cytokines). We therefore analyzed the data twice using each of these groupings.

**Figure 1:**
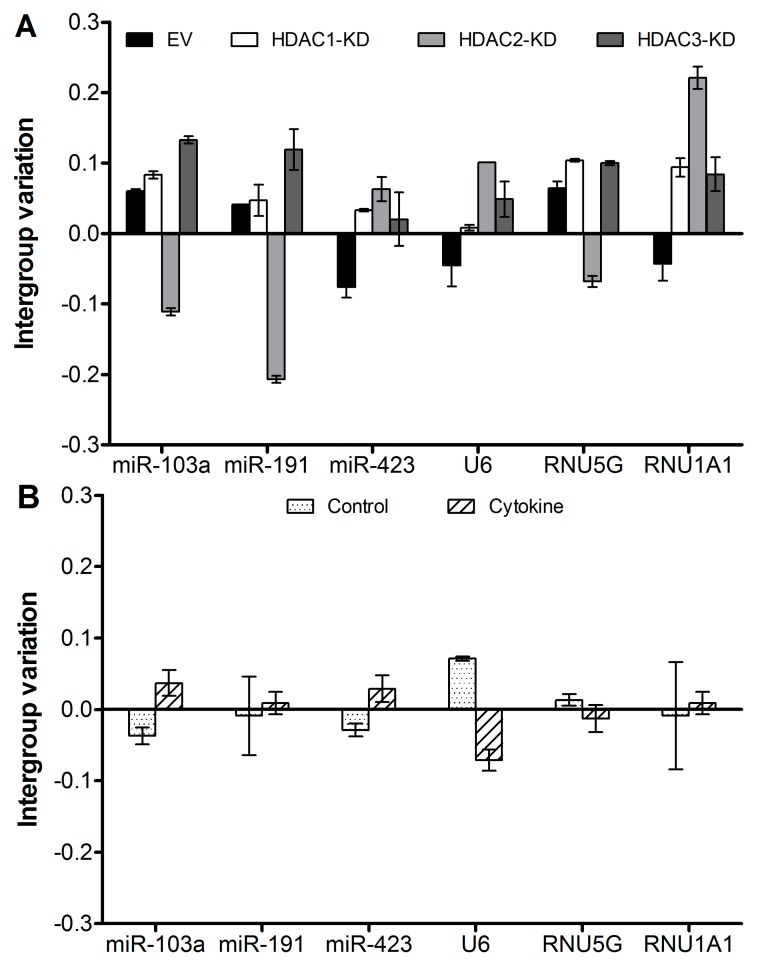
Intergroup variation of expression of six reference mRNAs/miRs in the array. Intragroup variation is given as error bars. Independent biological triplicates from each of the four cell lines were exposed cytokines or control medium for 6 h. RNA was purified, and triplicates were pooled and analyzed by two technical array replicates. Hence, data from eight samples were analyzed by NormFinder and divided by transduced cell line into four groups of 2n (**A**) or by exposure to cytokines or control medium into two groups of 4n (**B**).

The NormFinder output is provided as arbitrary “stability values”, where the smallest stability value signifies the highest stability. The stability values take into account the *intergroup variation*, which provides a measure of how much bias is introduced by normalizing to the given reference and of the *intragroup variation*, *i.e.*, the confidence interval within each group. Figure 1 shows the intergroup variation of the reference candidates from the array as confidence intervals, and Table 2 shows the stability values. As seen in Figure 1B, the intragroup variation can be larger than the intergroup variation, indicating either that expression levels are not very stable within the group or that the variation between the groups is even lower than the normal intragroup variation. As all values are arbitrary, it is not possible to discriminate between these two possibilities. However, this is subordinate, as both types of variation are accounted for when the NormFinder algorithm calculates the stability value.

Figure 1 and Table 2 show that the stabilities of the six candidates are in a similar range. Of note, there are both miR and non-miR references among these six candidates, which does not seem to influence the stability. We suggest that it is preferable to use miR for normalization of miR quantification. Thereby, the targets and references can be characterized in parallel. If the techniques used should include any bias against miRs in general, the reference miR will be affected similarly.

### 2.3. Stability Analysis of miR for Normalization of qPCR Quantification of Specific miR Expression

**Figure 2:**
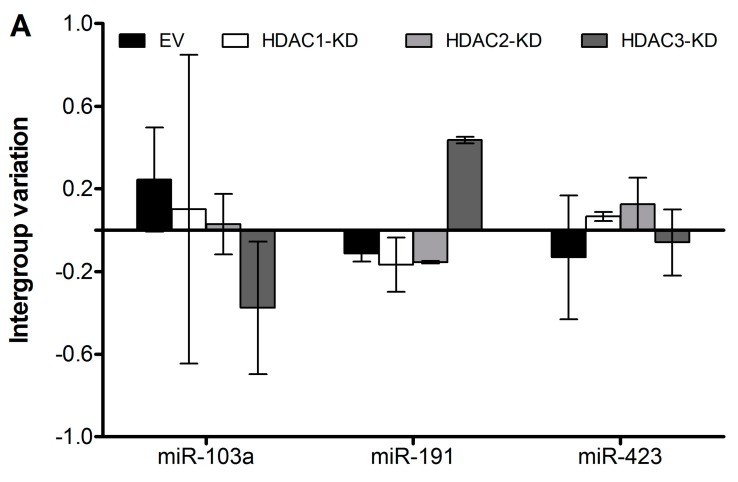

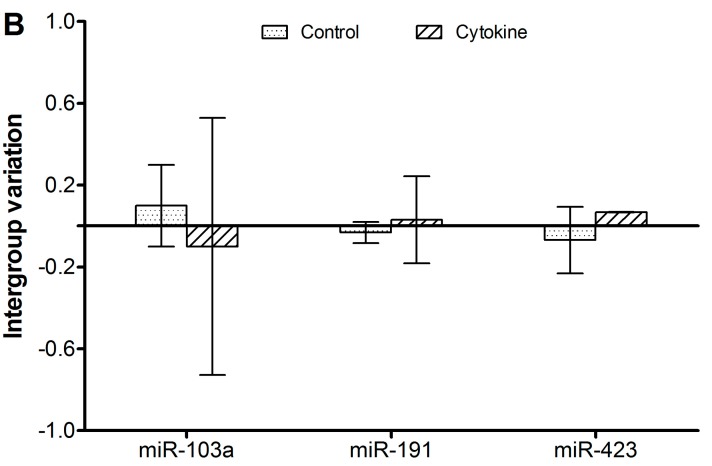
Intergroup variation of three reference miRs quantified by qPCR. Intragroup variation is given as error bars. One sample from each of the four cell lines were exposed to cytokines for 6–24 h or control medium. RNA was purified, and samples were analyzed by qPCR. In total, 24 samples were analyzed by NormFinder, divided by cell line into four groups of 6n (**A**) or by exposure into two groups of 12n (**B**).

We next measured the expressional levels of the three reference miRs by specific qPCR and analyzed their stability with NormFinder, as shown in Figure 2 and Table 3. The stability values depict that miR-423 is the most stable of the three. However, it is recommended that more than one reference gene for normalization is used [2,3,4], and NormFinder also gives the stability value of the best combination of two genes (Table 3). Note that this might not include the miR with the lowest stability value, because normalization to the combination of two other miRs might compensate for the bias introduced by normalization to the individual miR. In this case, the best combination of two reference miRs differs according to how the samples are grouped, and the user must then make the choice depending on the research hypothesis in question.

### 2.4. Stability Ranking of All miR in qPCR-Based miR Array

Finally, we ranked all miR quantified in the array using NormFinder, of which the top ten and the bottom-ranking miRs are given in Table 4. This shows that the difference between the most stable and the least stable miRs is very large when including all miRs, obviously including highly regulated miRs. Notably, two of the three miRs selected for this study, namely miR-103a and miR-423, figure in the top ten ranking miRs of the array in both groupings. This confirms that these two miRs are indeed stably expressed in both the array and specific qPCR quantifications, and a combination of these two miRs is appropriate for normalizing qPCR data from INS-1 cells in the conditions used here.

## 3. Discussion

In this study, we systematically analyzed the stability of several reference mRNAs and miRs for use in qPCR-based quantification of candidate miRs from INS-1 cells. Insulin-producing cell lines provide the advantage of representing only one islet cell subtype, but differ in many respects from primary islet β-cells. Further, since primary islets contain non-β endocrine and non-endocrine passenger cells, the RNA purification procedure for INS-1 cells may need separate optimization when isolating miR from intact islets.

For INS-1 cells, our efforts resulted in the choice of a normalization factor based on miR-103a and miR-423 for this particular setup, but this work more importantly outlines a broadly applicable example on how to choose the most stable miR(s) for normalization in relative quantification of specific qPCR. The same method can be applied for array-based expressional profiling. However, in most cases, using a global normalization procedure, such as the quantile method or the geometric mean, is more appropriate for this experiment type [4,17]. Nonetheless, analyzing the stability of a broad range of miRs from array data functions well as a screening method when searching for stably expressed miRs in a given experimental setup.

## 4. Materials and Methods

The rat insulinoma-derived β-cell line INS-1 is a standard model for studying β-cell function due to its responsiveness to glucose and degree of differentiation [13]. The INS-1 cell line was a generous gift from Claes Wollheim (Department of Cell Physiology and Metabolism, University Medical Center, Geneva, Switzerland). INS-1 cell lines with stable lentiviral transduction of shRNAs entailing knockdowns of HDAC1, HDAC2, and HDAC3 or a mock transduction with the empty vector construct (EV) were produced, and knockdown was verified by real-time qPCR and Western blotting [14]. Cells were maintained in RPMI-1640 medium with GlutaMAX, supplemented with 10% fetal calf serum, 100 U/mL penicillin, 10 μg/mL streptomycin, 50 μM β-mercaptoethanol, and 2.5 μg/mL puromycin. The cells were cultured at 37 °C in a humidified atmosphere containing 5% CO_2_. Medium changes and cell passage were performed weekly. Cells were seeded in 6-well plates (1.5 mio cells/well) and left for two days prior to exposure to 150 pg/mL IL-1β and 0.1 ng/mL IFNγ for 6 h before harvest. The cells were lysed and microRNA-enriched total RNA purified according to the kit manufacturer’s protocols.

For microRNA array analysis, the SYBR Green-based microRNA Ready-to-Use PCR Panels (Exiqon, Vedbaek, Denmark) were used according to the manufacturer’s protocols.

For SYBR Green-based qPCR quantification of specific miRs, the Universal cDNA Synthesis Kit II and the ExiLENT SYBR^®^ Green master mix (Exiqon) were used according to the manufacturer’s protocols with the following modifications based on careful optimizations: 20 ng/μL total RNA in 10-μL cDNA reactions and 40× cDNA dilutions in 10-μL qPCR reactions were used. Primer efficiencies were all in the range of 90%–105%.

For NormFinder analysis, software download and detailed description of use and data interpretation can be found at [18].

## 5. Conclusions

A small effort in optimizing the normalization of the relative quantification of miRs will enhance data validity and is recommended for all studies.

## Figures and Tables

**Table 1:** Concentrations, purity, and integrity of RNA from three purification kits.

RNA Purification Kit	Sample	Concentration (ng/µL)	OD 260/230	OD 260/280	RIN
miRCURY cell & plant (Exiqon)	1	263	1.7	2.0	9.5
	2	238	1.9	2.0	8.1
	3	299	2.0	2.0	9.7
	4	307	2.1	2.0	9.3
NucleoSpin miRNA (Macheray–Nagel)	1	111	2.2	2.1	6.8
	2	117	1.9	2.1	9.7
	3	167	1.8	2.1	9.6
	4	353	1.4	2.0	7.3
miRNeasy (Qiagen)	1	131	1.4	1.9	9.4
	2	134	0.3	2.0	9.5
	3	145	0.8	2.0	3.9
	4	184	1.9	2.0	9.5

RNA concentration and optical density (OD) ratios, an indicator of purity, were measured by NanoDrop and RNA Integrity Values (RIN) by BioAnalyzer (Agilent Technologies, Santa Clara, CA, USA).

**Table 2:** Stability values of six reference genes/miRs in the array.

Gene Name	Average *C*_t_ ± SD	Stability Value (A, Cell Line Grouping)	Stability Value (B, Exposure Grouping)
rno-miR-103a	25.6 ± 0.3	0.134	0.045
rno-miR-191	29.1 ± 0.4	0.151	0.067
rno-miR-423	30.3 ± 0.2	0.119	0.043
U6	21.4 ± 0.2	0.111	0.035
RNU5G	20.0 ± 0.3	0.126	0.042
RNU1A1	18.1 ± 0.2	0.172	0.074

Data obtained as given in legend to Figure 1. The smaller the stability value, the more stable the expression is.

**Table 3:** Stability values of three reference miR quantified by qPCR.

miR Name	Average *C*_t_ ± SD	Stability Value (A, Cell Line)	Stability Value (B, Treatment)
miR-103a	35.0 ± 0.7	0.380	0.179
miR-191	34.3 ± 0.9	0.303	0.099
miR-423	23.6 ± 0.7	0.255	0.063
Best combination of two genes		miR-103a and miR-191	miR-191 and miR-423
Stability value for best combination of two genes		0.258	0.067

Data obtained as given in the legend of Figure 2. The smaller the stability value, the more stable the expression is.

**Table 4:** Ranking of miR quantified by array according to stability value.

Cell Line Grouping			Exposure Grouping		
Rank	miR Name	Stability Value	Rank	miR Name	Stability Value
1	rno-miR-132	0.066	1	rno-let-7b	0.049
2	rno-let-7b	0.080	2	rno-miR-103a	0.053
3	rno-miR-30c	0.089	3	mmu-miR-671-5p	0.054
4	mmu-miR-671-5p	0.093	4	rno-miR-132	0.058
5	rno-miR-130a	0.095	5	rno-miR-331	0.058
6	rno-miR-103a	0.096	6	mmu-miR-1195	0.062
7	mmu-miR-1195	0.099	7	rno-miR-15b	0.065
8	rno-miR-204	0.099	8	rno-miR-125a-5p	0.068
9	rno-miR-423	0.100	9	mmu-let-7g	0.070
10	rno-miR-15b	0.109	10	rno-miR-423	0.070
416	mmu-miR-155	0.882	416	mmu-miR-155	1.475
